# Plasma Rich in Growth Factors as an Adjuvant Agent in Non-Penetrating Deep Sclerectomy

**DOI:** 10.3390/jcm12103604

**Published:** 2023-05-22

**Authors:** Pedro P. Rodríguez-Calvo, Ignacio Rodríguez-Uña, Andrés Fernández-Vega-Cueto, Ronald M. Sánchez-Ávila, Eduardo Anitua, Jesús Merayo-Lloves

**Affiliations:** 1 Instituto Universitario Fernandez-Vega, Fundación de Investigación Oftalmológica, University of Oviedo, 33012 Oviedo, Spain; 2 Instituto de Investigación Sanitaria del Principado de Asturias, 33011 Oviedo, Spain; 3 Biotechnology Institute (BTI), 01007 Vitoria, Spain; 4 Regenerative Medicine Laboratory, University Institute for Regenerative Medicine and Oral Implantology (UIRMI), 01007 Vitoria, Spain

**Keywords:** glaucoma filtration surgery, non-perforating deep sclerectomy, open-angle glaucoma, plasma rich in growth factors, PRGF, surgery outcome, plasma rich in growth factors, immunosafe eye drops, is-ePRGF

## Abstract

Background: The purpose of this study is to evaluate the utility and safety of plasma rich in growth factors immunosafe eye drops (is-ePRGF) in the postoperative treatment of non-penetrating deep sclerectomy (NPDS). Methods: This is a case–control study in patients with open-angle glaucoma. Group one (control) was not treated with is-ePRGF, while group two (is-ePRGF) was treated (four times a day for four months). Postoperative evaluations were performed at one day, one month, three months and six months. The main outcomes were: intraocular pressure (IOP), microcysts in blebs with AS-OCT and the number of hypotensive eye drops. Results: Preoperatively, group one (*n* = 48 eyes) and group two (*n* = 47 eyes) were similar in age (71.5 ± 10.7 vs. 70.9 ± 10.0 years; *p* = 0.68), IOP (20.6 ± 10.2 vs. 23.0 ± 9.0 mmHg; *p* = 0.26) and number of hypotensive drugs (2.7 ± 0.8 vs. 2.8 ± 0.9; *p* = 0.40). The IOP at six months dropped to 15.0 ± 8.0 mmHg (IOP reduction: −27.2%) and 10.9 ± 4.3 mmHg (IOP reduction: −52.6%) for group one and group two, respectively (*p* < 0.01). At six months, blebs with microcysts were 62.5% (group one) and 76.7% (group two). Postoperative complications were observed in 12 eyes (25%) for group one and in 5 eyes (11%) for group two (*p* = 0.06). No specific complications related to the use of is-ePRGF were identified. Conclusions: Topical is-ePRGF seems to reduce IOP and the rate of complications in the medium term after NPDS, so it can be considered as a possible safe adjuvant to achieve surgical success.

## 1. Introduction

Non-penetrating deep sclerectomy (NPDS) is a safe and effective option for intraocular pressure (IOP) reduction in glaucoma patients with a rapid disease progression, high IOP values or poor response to medical therapy [1,2,3]. The long-term success of this filtering surgery depends, in part, on maintaining adequate bleb morphology. Indeed, one of the leading causes of failure in this surgical technique is excessive scarring in the subconjunctival area or the intra-scleral space, causing fibrotic adhesions which compromise aqueous humor drainage and, consequently, increase IOP [1,4,5]. Therefore, prevention and postoperative management of fibrosis would determine postoperative outcomes, in terms of IOP and surgical success rate.

Intraoperative mitomycin C (MMC) and postoperative topical corticosteroids are widely used to prevent bleb fibrosis. Guedes et al. [4] reported that the use of MMC increased the success rate by 2.4-fold compared with not using it. Despite applying intraoperative MMC, postoperative bleb manipulations are often required to maintain IOP control [1]. Therefore, it is of interest to explore adjuvant therapeutic approaches to MMC to preserve bleb morphology and reduce the postoperative scarring response, aiming to promote long-term IOP control and decrease the incidence of postoperative bleb manipulations. 

Plasma rich in growth factors (PRGF) is a blood derivative product of autologous origin with important biological features such as antimicrobial, anti-inflammatory and anti-fibrotic properties. PRGF, in its different therapeutic formulations (eye drops, clot and membrane), has been effectively used to treat several ocular surface and corneal diseases [6,7] and even in macular-hole surgery [8,9], demonstrating its role in tissular regeneration, inflammation control and fibrosis modulation [10,11]. The biological properties of PRGF would theoretically be of great help in reducing fibrosis after NPDS. Rodriguez-Agirretxe et al. [12], in a pilot study, evaluated a case series of 10 eyes that underwent NPDS with a PRGF clot inserted in the subconjunctival space. The authors concluded that PRGF might enhance surgery success rates and reduce the need for postoperative medications. Furthermore, they also pointed out that postoperative treatment with PRGF eye drops could improve the results.

The present study aimed to evaluate the potential benefits of the coadjuvant postoperative treatment with PRGF eye drops in patients who underwent NPDS with MMC.

## 2. Materials and Methods

This study included patients diagnosed with primary open-angle glaucoma (POAG) with uncontrolled IOP (≥22 mm Hg), even with maximal tolerated medical therapy or laser trabeculoplasty, who underwent NPDS. Exclusion criteria were: age < 18 years, treatment with PRGF over the 12 months previous to NPDS surgery, previous glaucoma surgery, severe ocular surface disease, infectious illness (HIV, HBV, HDV, syphilis) and pregnancy or lactation. The study was approved by the Ethics Committee of the ‘Principado de Asturias’ (Oviedo, Spain) and adhered to the tenets of the Declaration of Helsinki. All the patients signed a consent form providing their approval for glaucoma surgery and blood extraction for PRGF elaboration.

The patients were divided into two groups: group 1 (control) corresponded to patients operated with NPDS who received standard postoperative treatment and did not receive PRGF eye drops as adjuvant treatment after surgery; these patients were selected retrospectively and sequentially immediately before the inclusion of the patients in group 2. Group 2 corresponded to the prospective cohort of patients who received standard postoperative treatment associated with PRGF eye drops as adjuvant therapy. Fifty eyes were included in each treatment group, which had to be followed up to 6 months after surgery.

### 2.1. Surgical Technique

All procedures were performed under peribulbar anesthesia by the same surgeon (P.P.R.-C.). A fornix-based peritomy was performed using a 7-0 silk corneal traction suture with conjunctival pocket dissection, followed by gentle cautery to achieve hemostasis. A 5 × 5 mm partial thickness superficial scleral flap was dissected (extended 1–2 mm into clear cornea) and 0.02% mitomycin C (MMC) was applied for 2 min between the sclera and conjunctiva. Then, the area was irrigated thoroughly with a balanced salt solution. Resection of a 4 × 4 mm deep scleral flap was performed, followed by trabecular-Descemet membrane (TDM) dissection and resection of the Schlemm’s canal using Mermoud forceps. A supraciliary pocket was made with a 45° blade for the incision and a blunt spatula, 2 mm behind the scleral spur, following the technique first described by Muñoz (2009) [13], and a hema implant (either Esnoper^®^ V-2000 or Esnoper^®^ Clip, AJL Ophthalmic S.A., Miñano, Alava, Spain) was placed inside [14]. The superficial scleral flap was reflected back without sutures and conjunctiva was closed with interrupted 10-0 nylon sutures. In the postoperative follow-up, a goniopuncture with Nd-Yag laser was performed when insufficient filtration was observed through the TDM [15].

### 2.2. PRGF Preparation

The PRGF elaboration was developed following the steps already published by our group elsewhere [16]. Before surgery, during the anesthetic process, blood from patients was collected into 9 mL tubes. Samples were centrifuged at 580× *g* for 8 min at room temperature in an Endoret System centrifuge (BTI Biotechnology Institute, S.L., Miñano, Alava, Spain). The whole column of PRGF was collected after centrifugation, avoiding the buffy coat that contains the leukocytes, using an Endoret ophthalmology kit (BTI Biotechnology Institute, S.L., Miñano, Spain). The obtained supernatant was incubated at 37 °C for 1 h and then heat treated at 56 °C for 60 min. The plasma supernatants were filtered, aliquoted and stored at −20 °C until use. 

Highly sterile conditions were followed for all procedures, operating inside a laminar flow hood. In order to inactivate the complement, the PRGF eye drops were heated to 57 °C; the resulting product is called immunosafe eye drops (is-ePRGF). Patients were instructed to keep the PRGF eye-drop dispensers at −20 °C; each dispenser was used for 3 consecutive days (the eye drops could be at 8 °C or ambient temperature).

### 2.3. Postoperative Treatment

Standard postoperative treatment consisted of moxifloxacin eye drops 4 times a day for one week, and dexamethasone eye drops every 2 h for one week and then in tapering frequency over the following 10 weeks after surgery: 5 times a day for 2 weeks, 4 times a day for 2 weeks, 3 times a day for 2 weeks, twice a day for 2 weeks and once a day for 2 weeks. Group 1 and group 2 patients received standard postoperative treatment. Patients in group 2 were additionally treated with is-ePRGF 4 times a day for 4 months (uninterruptedly). The is-ePRGF were prescribed to be used 4 times a day distributed throughout the day, approximately every 6 h, leaving a 5 min interval among the other drops. In the hypothetical case of loss or completion, a new blood test was performed at one of the routine follow-up visits to prevent the patient from running out of treatment.

### 2.4. Study Variables

Follow-up visits were at 1 day and 1-, 3- and 6 months after NPDS. The variables analyzed included IOP measurement, bleb height and presence of microcysts in bleb (Anterior Segment Optical Coherence Tomography; AS-OCT, CASIA2, Tomey, Japan), the incidence of complications and frequency of bleb manipulations. The measurements and assessment of AS-OCT parameters were carried out by one observer (P.P.R.-C.) masked to the IOP level and surgical outcome.

Furthermore, the surgical outcomes were stratified into three levels: (1) complete success, defined as having an IOP ≤ 21 mmHg without antiglaucoma medications; (2) qualified success, defined as having an IOP ≤ 21 mmHg with antiglaucoma medications; (3) failure, defined as having IOP ≤ 6 mmHg in two consecutive visits, additional glaucoma surgeries (needling was not considered as secondary glaucoma surgery), or not achieving the qualification of complete or qualified success.

### 2.5. Statistical Analisys

Data analysis was performed using SPSS for Windows, version 25.0 (SPSS Inc., Chicago, IL, USA). Normality was checked by means of the Kolmogorov–Smirnov test. A descriptive analysis of the sociodemographic variables expresses the mean values with their standard deviation. The absolute and relative frequencies were determined for categorical variables, and for continuous variables, the mean, standard deviation, minimum and maximum were calculated. For comparisons between pre-and post-treatment values, parametric (Student’s t or ANOVA) or non-parametric (Mann–Whitney or Kruskal–Wallis) tests are used in the case of continuous variables, and for categorical variables, the chi-square test was used. Continuous variables were analyzed using repeated-measures analysis of variance (ANOVA). Bonferroni test was performed to analyze significant differences for the variables throughout the time within a group, while differences between two groups at each visit were analyzed with the independent-sample *t*-test. Fisher exact test was used to compare categorical data between both studied groups. Differences were considered statistically significant when the *p* value was <0.05.

## 3. Results

Forty-eight eyes were included in group one (control) and forty-seven in group two (is-ePRGF). The detailed characteristics of the patients before surgery are shown in Table 1. Among the 50 selected controls (group one), there were two losses: one died during follow-up, and one did not attend scheduled appointments. In group two, three withdrawals of the fifty selected cases that had signed the informed consent were reported: one patient requested to leave the study by their own decision, and in the other two cases the sample was not valid to prepare the is-ePRGF. No differences were found in the demographic data or the preoperative clinical characteristics between the two groups studied (Table 1).

**Figure 1:**
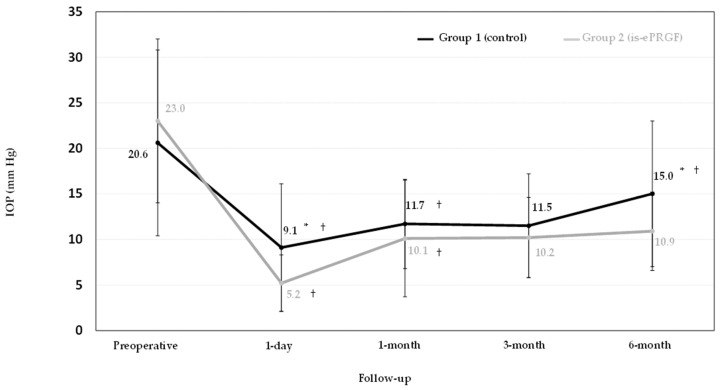
Measurement of intraocular pressure after surgery. *: Differences between groups at the follow-up visit. †: Differences with previous visit within the group. is-ePRGF: immunosafe eye drops plasma rich in growth factors; IOP: intraocular pressure.

NPDS showed a significant reduction in IOP at each postoperative follow-up visit concerning preoperative values in both groups. In group one (control), there was a significant IOP increase between 3- and 6-month follow-up visits (*p* = 0.01). In group two (is-ePRGF), IOP was maintained from the first month throughout the follow-up period (*p* = 0.15). At the last follow-up visit, the IOP was significantly lower in group two than that in group one (*p* < 0.01) (Figure 1). The IOP reduction at 6 months compared with that preoperatively was 27.2% and 52.6% in group one and group two, respectively. In a further analysis, each patient’s IOP reduction was calculated, showing mean values of 11.0 ± 51.5% in group one and 34.5 ± 30.9% in group two (*p* < 0.01). The number of hypotensive medications significantly decreased to 0.3 ± 0.9 and 0.2 ± 0.6 in group one and group two, respectively (*p* < 0.01).

**Figure 2:**
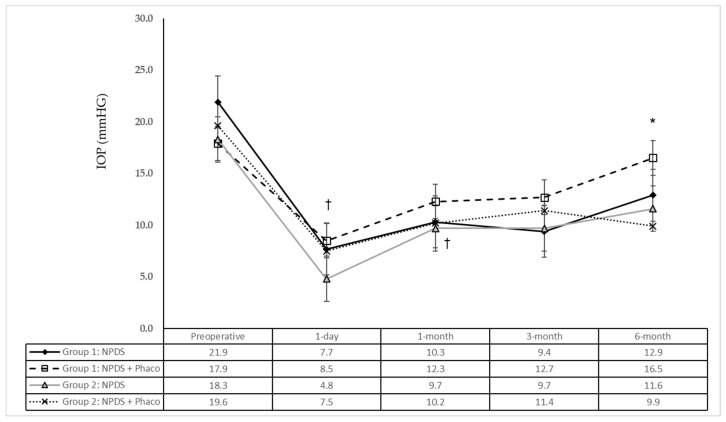
Measurement of intraocular pressure after surgery. *: Differences between groups at the follow-up visit. †: Differences with previous visit within the group. IOP: intraocular pressure; NPDS: non-penetrating deep sclerectomy; Phaco: phacoemulsification and intraocular lens implantation.

Further comparisons according to the surgical technique (Figure 2) showed statistically significant differences at 6 months between controls that underwent NPDS + Phaco and NPDS + Phaco in the is-ePRGF group (*p* = 0.01). IOP was also lower in NPDS with is-ePRGF compared with that in NPDS in controls (*p* = 0.26).

The percentages of complete and qualified success at the end of the follow-up were 66.7% and 83.3%, respectively, in group one, and 72.3% and 93.6% in group two. A further analysis by subgroups is shown in Table 2: the subgroup of NPDS + phaco treated with is-ePRGF presented the lowest failure rate (6.3%), followed by the subgroup of NPDS with is-ePRGF (6.5%). 

**Figure 3:**
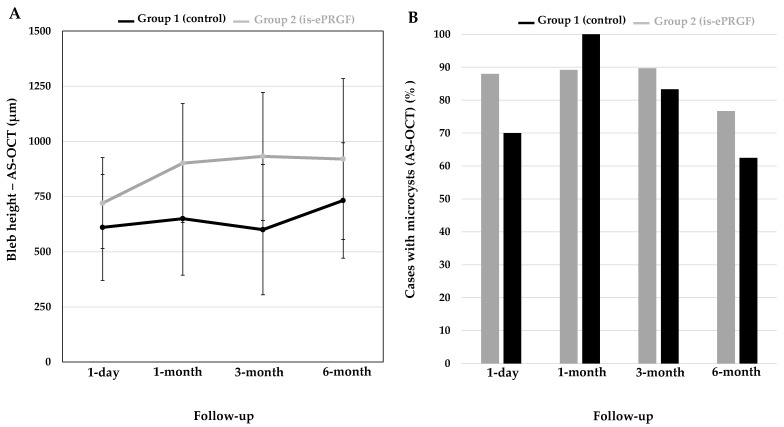
Measurement of bleb with AS-OCT. (**A**) Bleb height; (**B**) frequency of microcysts. is-ePRGF: immunosafe eye drops plasma rich in growth factors.

The bleb evaluation with AS-OCT revealed a higher bleb height at all follow-up visits in group two compared with that in group one. In group two, an increase in bleb height was observed between 1 day and 1 month, and then it remained stable (Figure 3A). At the final visit, the percentages of blebs presenting microcysts were 62.5% and 76.7% in group one and group two, respectively (Figure 3B).

Table 3 shows the postoperative complications and frequency of bleb manipulations in each group. There were no complications related to the use of is-ePRGF. Bleb manipulations (laser goniopuncture or needling) were performed in 39.6% and 38.3% of the eyes in group one and group two, respectively. Hypotensive medication was required in 29.2% of the cases in group one and in 21.3% of group two. Postoperative complications were more frequent in group one. In group one, 6.3% of the eyes required a secondary glaucoma surgery, and this was 2.1% in group two.

The requirement of hypotensive medications and further glaucoma surgery were both less frequent in group two, without reaching statistical significance (*p* = 0.42 and *p* = 0.78, respectively).

## 4. Discussion

NPDS is a filtering glaucoma surgery that emerged as an alternative to trabeculectomy in patients with open-angle glaucoma [2], providing satisfactory efficacy and a low rate of complications [3]. However, the surgical success rate (meaning a goal IOP within adequate limits) tends to decrease over the long-term [1,17]. Postoperative fibrosis is the leading cause of surgical failure [17,18]. Consequently, huge efforts are performed to find effective strategies to modulate the healing process. To this extent, the strategies may aim to act on the four wound-healing pathways, coagulative, inflammatory, proliferative and post-proliferative remodeling [19], and may be applied at three different stages: pre-, intra- and postoperatively. In the current study, we evaluated a strategy which consisted of reinforcing the conventional one (that is, intraoperative MMC [20,21] and postoperative steroids) with PRGF at the postoperative stage. PRGF has demonstrated its role in tissular regeneration, inflammation control and fibrosis modulation. Hence, it could act on the inflammatory, proliferative and post-proliferative remodeling of the wound-healing pathways. 

Our results showed that reinforcing the conventional strategy with postoperative is-ePRGF yielded a higher IOP reduction at 6 months postoperatively (27.2% vs. 52.6% of IOP reduction in the control and PRGF groups, respectively) and a lower percentage of failed surgical outcomes (16.7% vs. 6.4%). 

Furthermore, AS-OCT analysis of bleb morphologies showed a higher bleb height as well as a higher proportion of blebs presenting microcysts in the PRGF group than in the control group. AS-OCT has been widely used in the postoperative evaluation of conjunctival blebs of several glaucoma filtering techniques [22,23,24,25]. Several parameters, such as bleb thickness, bleb height, bleb wall reflectivity, the presence or absence of microcysts, etc., have been studied, looking for an association with better IOP control. In general, a tall bleb with a thick hypo-reflective wall is considered as a feature of a well-functioning bleb [26]. Hirooka et al. [27] studied trabeculectomy bleb images with time-domain OCT, looking for an association between morphological features and function. They found that the so-called cystoid-type blebs (characterized by the presence of multiple small cysts inside the bleb) were related to a higher probability of success compared with other filtering blebs presenting less cystic spaces. This is in accordance with other studies [28,29]. 

In addition, a higher proportion of blebs presenting microcysts as well as taller blebs were observed in the is-ePRGF group compared with the control group. These facts could be related with the lower IOP and the higher success rate in the is-ePRGF group at the final follow-up visit. Therefore, the better feature appearance of the bleb morphology in the is-ePRGF group would also support the benefits of using coadjuvant is-ePRGF eye drops in NPDS. 

To the best of our knowledge, this is the first study that evaluated coadjuvant postoperative treatment with PRGF immunosafe eye drops in patients who underwent NPDS. However, it is worth noting the study by Rodriguez-Agirretxe et al. [12], who also evaluated PRFG, although in another therapeutic formulation (fibrin membrane), and in another stage (intraoperatively). The IOP reduction in our study at the 6-month follow-up visit was comparable to that reported by Rodriguez-Agirretxe et al. [12] (around 50%); they extended the follow-up for two years, finding that IOP was stabilized at levels of approximately 15 mmHg. Furthermore, their complete and qualified success percentages were 80 and 90%, respectively, two years after NPDS. These results support the hypothesis of the benefit of PRGF for enhancing the surgical success rate of NPDS. 

The use of amniotic membrane (AM) has also been reported to be a safe and effective adjuvant treatment in glaucoma filtering surgery [30]. Similarly to the present study, Sheha et al. [31] reported that the use of single-layer AM under the scleral flap in trabeculectomy with MMC provided lower IOP, higher success rates and lower complication rates compared with trabeculectomy with MMC alone. However, the method of application of AM in filtering surgery (whether above or below the scleral flap) is yet to be standardized [32].

Despite the positive presented results in our study in terms of IOP control, surgical success rate and bleb features, it is interesting to note that there were no differences between groups in the rate of postoperative bleb manipulation, number of required hypotensive medications and necessity of secondary glaucoma surgery (Table 2). We hypothesized that depending on the formulation (eye drop or fibrin membrane), PRGF could have a different effect on NPDS surgery. In eye drops, the percentage of penetration reaching the bleb would be lower than the fibrin membrane. Consequently, its impact on modulating bleb fibrosis would be limited, and it would explain the absence of differences between the control and PRGF groups in postoperative management. By contrast, the use of postoperative PRGF eye drop formulation, beyond the moderate healing process in the bleb (which could depend on the degree of PRGF eye drop penetration), could contribute to other properties to improve IOP control after NPDS. Firstly, the PRGF eye drop itself could decrease the IOP by modulating the transforming growth factor-β (TGF-β) activity [16]. The increased activity of TGF-β may provide an accumulation of deposits in the extracellular matrix of the trabecular tissue, limiting the aqueous humor flow through the trabecular meshwork and causing an IOP rise [33,34]. Consequently, the capability of PRGF eye drops to modulate the TGF-β activity would act as hypotensive and could explain the lower postoperative IOP in the PRGF group. Secondly, for those patients who required postoperative hypotensive medication, the anti-inflammatory properties of the PRGF eye drop could contribute to higher treatment adherence, augmenting the hypotensive effect and increasing the rate of qualified success (defined as having an IOP ≤ 21 mmHg with antiglaucoma medications). 

There were no PRGF-related complications over the follow-up. Of note that in this study, we evaluated the postoperative PRGF eye drop combined with intraoperative MMC and postoperative steroids. The absence of potential complications associated with PRGF eye drops is another crucial aspect owing to be combined with any of the strategies studied to modulate wound healing of filtering blebs, such as preoperative steroids, intraoperative antifibrotics, anti-VEGF, PRGF membrane, or amniotic membrane. Furthermore, we observe a decrease in the rate of postoperative complications in the PRGF group compared with the control group.

Despite these encouraging outcomes, it should be noted that our study has limitations, such as the retrospective analysis in the control group and a short follow-up. In addition, the postoperative AS-OCT evaluation did not include bleb wall reflectivity assessment, which might be related to bleb functionality [22,25,26]. Finally, the relatively small sample size within some subgroups might imply that some results need to be analyzed with caution. Ideally, in the design of future prospective randomized clinical trials, a larger number of patients undergoing only one type of surgical technique may be selected in order to reduce the risk of bias.

## 5. Conclusions

The results of this study would suggest that PRGF immunosafe eye drops may be considered as a safe non-invasive adjuvant agent in the postoperative treatment of NPDS. Further prospective and randomized clinical trials should be performed to confirm the potential therapeutic efficacy of PRGF eye drops in glaucoma surgery.

## Figures and Tables

**Table 1:** Baseline demographics and preoperative clinical characteristics by subgroups.

Parameter	Group 1 (Control) *n* = 48		Group 2 (Is-ePRGF) *n* = 47		*p* Value
	NPDS	NPDS + Phaco	NPDS	NPDS + Phaco	
Eyes (*n*)	19	29	31	16	
Age (years)	72.7 ± 8.4	70.7 ± 12.0	69.7 ± 11.1	69.6 ± 7.5	0.73
	(58–91)	(34–88)	(51–91)	(52–80)	
Sex (Female/Male)	10/9	12/17	18/13	6/10	0.46
	(52.6%/47.4%)	(41.4%/58.6%)	(58.1%/41.9%)	(37.5%/62.5%)	
CDVA (logMAR)	0.39 ± 0.38	0.25 ± 0.44	0.27 ± 0.27	0.10 ± 0.14	0.01 *
	(1.00–0.00)	(1.70–0.00)	(1.30–0.00)	(0.40–0.00)	
IOP (mmHg)	21.9 ± 12.6	17.9 ± 7.3	18.3 ± 6.8	19.6 ± 7.1	0.79
	(9.0–50.0)	(10.0–44.0)	(8.0–37.0)	(11.0–35.0)	
Eyes with glaucoma eyedrop treatment	19 (100%)	29 (100%)	31 (100%)	16 (100%)	
No. of hypotensive medications	2.6 ± 0.6	2.9 ± 0.8	2.9 ± 0.9	2.8 ± 0.8	0.44
Visual field mean deviation (dB)	−18.7 ± 7.2	−13.5 ± 7.7	−16.1 ± 9.2	−15.0 ± 7.7	0.21
Glaucoma severity					
*Early*	1 (5.3%)	6 (20.7%)	5 (16.1%)	2 (12.5%)	0.42
*Moderate*	3 (15.8%)	8 (27.6%)	6 (19.4%)	7 (43.8%)	
*Advanced*	14 (73.7%)	12 (41.4%)	15 (48.4%)	7 (43.8%)	
*Terminal*	1 (5.3%)	3 (10.3%)	5 (16.1%)	−	
Type of glaucoma, *n* (%)					
*POAG*	4 (21.1)	12 (41.4)	8 (25.8)	5 (31.3)	0.50
*Glaucoma and high myopia*	6 (31.6)	3 (10.3)	11 (35.5)	−	
*PXFG*	5 (26.3)	11 (37.9)	9 (29.0)	7 (43.8)	
*UG*	1 (5.3)	1 (3.4)	−	1 (3.2)	
*NTG*	2 (10.5)	1 (3.4)	1 (3.2)	1 (3.2)	
*TG*	1 (5.3)	−	1 (3.2)	1 (3.2)	
*PG*	–	1 (3.4)	1 (3.2)	1 (3.2)	

Data presented as mean ± SD (range) or No. (%). * NPDS group 1 vs. NPDS + Phaco group 2. is-ePRGF: immunosafe eye drops plasma rich in growth factors; CDVA: corrected distance visual acuity; IOP: intraocular pressure; NPDS: non-penetrating deep sclerectomy; NTG: normal-tension glaucoma; PG: pigmentary glaucoma; Phaco: Phacoemulsification and intraocular lens implantation; POAG: primary open angle glaucoma; PXFG: pseudoexfoliative glaucoma; TG: secondary open angle glaucoma due to ocular trauma; UG: uveitic glaucoma.

**Table 2:** Success rates at 6 months (intraocular pressure ≤ 21 mm Hg) by subgroups.

	Group 1 (Control)		Group 2 (Is-ePRGF)	
	NPDS	NPDS + Phaco	NPDS	NPDS + Phaco
	(*n* = 19)	(*n* = 29)	(*n* = 31)	(*n* = 16)
Complete success, *n* (%)	14 (73.7)	18 (62.1)	21 (67.7)	13 (81.3)
Qualified success (complete success + success with treatment), *n* (%)	16 (84.2)	24 (82.8)	29 (93.5)	15 (93.8)
Failure, *n* (%)	3 (15.8)	5 (17.2)	2 (6.5)	1 (6.3)

**Table 3:** Comparison of bleb manipulations, hypotensive medication, postoperative complications and secondary glaucoma surgery between groups.

Parameter	Group 1 (Control)	Group 2 (Is-ePRGF)	*p* Value
Bleb manipulation	19 (39.6%)	18 (38.3%)	0.58
Laser goniopuncture	14 (29.2%)	10 (21.3%)	0.36
Needling	5 (10.4%)	8 (17.0%)	0.58
Hypotensive medications	14 (25.0%)	10 (21.3%)	0.42
Postoperative complications	12 (25.0%)	5 (10.6%)	0.06
Hyphema	4 (8.3%)	2 (4.3%)	
Atalamia	2 (4.2%)	0 (0.0%)	
Hypotony	3 (6.3%)	1 (2.1%)	
Iris incarceration	2 (4.2%)	0 (0.0%)	
TDM rupture	1 (2.1%)	2 (4.3%)	
Secondary glaucoma surgery	3 (6.3%)	1 (2.1%)	0.78
Bleb revision	2 (4.2%)	1 (2.1%)	
GDD implantation	1 (2.1%)	0 (0.0%)	

GDD: Glaucoma drainage device; TDM: Trabecular-Descemet membrane.

## Data Availability

The data used to support this study’s findings are available upon request to the corresponding author.

## References

[1] Shaarawy T., Mansouri K., Schnyder C., Ravinet E., Achache F., Mermoud A. (2004). Long-term results of deep sclerectomy with collagen implant. J. Cataract Refract. Surg..

[2] Mendrinos E., Mermoud A., Shaarawy T. (2008). Nonpenetrating glaucoma surgery. Surv. Ophthalmol..

[3] Eldaly M.A., Bunce C., Elsheikha O.Z., Wormald R. (2014). Non-penetrating filtration surgery versus trabeculectomy for open-angle glaucoma. Cochrane Database Syst. Rev..

[4] Guedes R.A.P., Guedes V.M.P., Chaoubah A. (2011). Factors associated with non-penetrating deep sclerectomy failure in controlling intraocular pressure. Acta Ophthalmol..

[5] Roy S., Mermoud A. (2006). Complications of deep nonpenetrating sclerectomy. J. Fr. Ophtalmol..

[6] Sanchez-Avila R.M., Merayo-Lloves J., Fernandez M.L., Rodriguez-Gutierrez L.A., Jurado N., Muruzabal F., Orive G., Anitua E. (2018). Plasma Rich in Growth Factors for the Treatment of Dry Eye after LASIK Surgery. Ophthalmic Res..

[7] Sanchez-Avila R.M., Merayo-Lloves J., Riestra A.C., Fernandez-Vega Cueto L., Anitua E., Begona L., Muruzabal F., Orive G. (2017). Treatment of patients with neurotrophic keratitis stages 2 and 3 with plasma rich in growth factors (PRGF-Endoret) eye-drops. Int. Ophthalmol..

[8] Sánchez-Ávila R.M., Robayo-Esper C.A., Villota-Deleu E., Fernández-Vega Sanz Á., Fernández-Vega González Á., de la Sen-Corcuera B., Anitua E., Merayo-Lloves J. (2022). Plasma Rich in Growth Factors in Macular Hole Surgery. Clin. Pract..

[9] Figueroa M.S., Mora Cantallops A., Virgili G., Govetto A. (2020). Long-term results of autologous plasma as adjuvant to pars plana vitrectomy in the treatment of high myopic full-thickness macular holes. Eur. J. Ophthalmol..

[10] Sanchez-Avila R.M., Merayo-Lloves J., Riestra A.C., Berisa S., Lisa C., Sanchez J.A., Muruzabal F., Orive G., Anitua E. (2018). Plasma rich in growth factors membrane as coadjuvant treatment in the surgery of ocular surface disorders. Medicine.

[11] Anitua E., de la Sen-Corcuera B., Orive G., Sánchez-Ávila R.M., Heredia P., Muruzabal F., Merayo-Lloves J. (2021). Progress in the use of plasma rich in growth factors in ophthalmology: From ocular surface to ocular fundus. Expert Opin. Biol. Ther..

[12] Rodríguez-Agirretxe I., Freire V., Muruzabal F., Orive G., Anitua E., Díez-Feijóo E., Acera A. (2018). Subconjunctival PRGF Fibrin Membrane as an Adjuvant to Nonpenetrating Deep Sclerectomy: A 2-Year Pilot Study. Ophthalmic Res..

[13] Muñoz G. (2009). Nonstitch suprachoroidal technique for T-flux implantation in deep sclerectomy. J. Glaucoma.

[14] Bonilla R., Loscos J., Valldeperas X., Parera M.A., Sabala A. (2012). Supraciliary hema implant in combined deep sclerectomy and phacoemulsification: One year results. Open Ophthalmol. J..

[15] Belda J.I., Loscos-Arenas J., Mermoud A., Lozano E., D’Alessandro E., Rebolleda G., Rodriguez-Agirretxe I., Canut M., Rodriguez-Calvo P.P. (2018). Supraciliary versus intrascleral implantation with hema implant (Esnoper V-2000) in deep sclerectomy: A multicenter randomized controlled trial. Acta Ophthalmol..

[16] Sánchez-Avila R.M., Merayo-Lloves J., Fernández M.L., Rodríguez-Gutiérrez L.A., Rodríguez-Calvo P.P., Fernández-Vega Cueto A., Muruzabal F., Orive G., Anitua E. (2018). Plasma rich in growth factors eye drops to treat secondary ocular surface disorders in patients with glaucoma. Int. Med. Case Rep. J..

[17] Bergin C., Petrovic A., Mermoud A., Ravinet E., Sharkawi E. (2016). Baerveldt tube implantation following failed deep sclerectomy versus repeat deep sclerectomy. Graefe’s Arch. Clin. Exp. Ophthalmol..

[18] Bissig A., Rivier D., Zaninetti M., Shaarawy T., Mermoud A., Roy S. (2008). Ten years follow-up after deep sclerectomy with collagen implant. J. Glaucoma.

[19] Fan Gaskin J.C., Nguyen D.Q., Soon Ang G., O’Connor J., Crowston J.G. (2014). Wound Healing Modulation in Glaucoma Filtration Surgery-Conventional Practices and New Perspectives: The Role of Antifibrotic Agents (Part I). J. Curr. glaucoma Pract..

[20] Cheng J.-W., Cai J.-P., Li Y., Wei R.-L. (2011). Intraoperative mitomycin C for nonpenetrating glaucoma surgery: A systematic review and meta-analysis. J. Glaucoma.

[21] Kozobolis V.P., Christodoulakis E.V., Tzanakis N., Zacharopoulos I., Pallikaris I.G. (2002). Primary deep sclerectomy versus primary deep sclerectomy with the use of mitomycin C in primary open-angle glaucoma. J. Glaucoma.

[22] Mastropasqua R., Fasanella V., Agnifili L., Curcio C., Ciancaglini M., Mastropasqua L. (2014). Anterior segment optical coherence tomography imaging of conjunctival filtering blebs after glaucoma surgery. Biomed Res. Int..

[23] Fernández-Buenaga R., Rebolleda G., Casas-Llera P., Muñoz-Negrete F.J., Pérez-López M. (2012). A comparison of intrascleral bleb height by anterior segment OCT using three different implants in deep sclerectomy. Eye.

[24] Aptel F., Dumas S., Denis P. (2009). Ultrasound biomicroscopy and optical coherence tomography imaging of filtering blebs after deep sclerectomy with new collagen implant. Eur. J. Ophthalmol..

[25] Pérez-Rico C., Gutiérrez-Ortíz C., Moreno-Salgueiro A., González-Mesa A., Teus M.A. (2014). Visante anterior segment optical coherence tomography analysis of morphologic changes after deep sclerectomy with intraoperative mitomycin-C and no implant use. J. Glaucoma.

[26] Narita A., Morizane Y., Miyake T., Seguchi J., Baba T., Shiraga F. (2018). Characteristics of early filtering blebs that predict successful trabeculectomy identified via three-dimensional anterior segment optical coherence tomography. Br. J. Ophthalmol..

[27] Hirooka K., Takagishi M., Baba T., Takenaka H., Shiraga F. (2010). Stratus optical coherence tomography study of filtering blebs after primary trabeculectomy with a fornix-based conjunctival flap. Acta Ophthalmol..

[28] Singh M., Chew P.T.K., Friedman D.S., Nolan W.P., See J.L., Smith S.D., Zheng C., Foster P.J., Aung T. (2007). Imaging of trabeculectomy blebs using anterior segment optical coherence tomography. Ophthalmology.

[29] Cerdà-Ibáñez M., Pérez-Torregrosa V.T., Olate-Pérez A., Almor Palacios I., Gargallo-Benedicto A., Osorio-Alayo V., Barreiro Rego A., Duch-Samper A. (2017). Qualitative analysis of repaired filtering blebs with anterior segment-optical coherence tomography. Arch. Soc. Esp. Oftalmol..

[30] Shen T.-Y., Hu W.-N., Cai W.-T., Jin H.-Z., Yu D.-H., Sun J.-H., Yu J. (2020). Effectiveness and Safety of Trabeculectomy along with Amniotic Membrane Transplantation on Glaucoma: A Systematic Review. J. Ophthalmol..

[31] Sheha H., Kheirkhah A., Taha H. (2008). Amniotic membrane transplantation in trabeculectomy with mitomycin C for refractory glaucoma. J. Glaucoma.

[32] Sharma R., Nappi V., Empeslidis T. (2023). The developments in amniotic membrane transplantation in glaucoma and vitreoretinal procedures. Int. Ophthalmol..

[33] Raychaudhuri U., Millar J.C., Clark A.F. (2018). Knockout of tissue transglutaminase ameliorates TGFβ2-induced ocular hypertension: A novel therapeutic target for glaucoma?. Exp. Eye Res..

[34] Agarwal R., Agarwal P. (2010). Future target molecules in antiglaucoma therapy: Tgf-Beta may have a role to play. Ophthalmic Res..

